# Spinal cord neurone loss and foot placement changes in a rat knock-in model of amyotrophic lateral sclerosis Type 8

**DOI:** 10.1093/braincomms/fcae184

**Published:** 2024-05-24

**Authors:** Brenda Murage, Han Tan, Tomoji Mashimo, Mandy Jackson, Paul A Skehel

**Affiliations:** Centre for Discovery Brain Sciences, Edinburgh University, Edinburgh EH8 9XD, UK; Euan MacDonald Centre for MND Research, Edinburgh University, Edinburgh EH16 4SB, UK; Centre for Discovery Brain Sciences, Edinburgh University, Edinburgh EH8 9XD, UK; Division of Animal Genetics, Laboratory Animal Research Center, Institute of Medical Science, The University of Tokyo, Tokyo 108-8639, Japan; Centre for Discovery Brain Sciences, Edinburgh University, Edinburgh EH8 9XD, UK; Euan MacDonald Centre for MND Research, Edinburgh University, Edinburgh EH16 4SB, UK; Centre for Discovery Brain Sciences, Edinburgh University, Edinburgh EH8 9XD, UK; Euan MacDonald Centre for MND Research, Edinburgh University, Edinburgh EH16 4SB, UK

**Keywords:** ALS8, motor neurone disease, VAPB

## Abstract

Amyotrophic lateral sclerosis is an age-dependent cell type–selective degenerative disease. Genetic studies indicate that amyotrophic lateral sclerosis is part of a spectrum of disorders, ranging from spinal muscular atrophy to frontotemporal dementia that share common pathological mechanisms. Amyotrophic lateral sclerosis Type 8 is a familial disease caused by mis-sense mutations in *VAPB*. VAPB is localized to the cytoplasmic surface of the endoplasmic reticulum, where it serves as a docking point for cytoplasmic proteins and mediates inter-organelle interactions with the endoplasmic reticulum membrane. A gene knock-in model of amyotrophic lateral sclerosis Type 8 based on the *VapB^P56S^* mutation and *VapB* gene deletion has been generated in rats. These animals display a range of age-dependent phenotypes distinct from those previously reported in mouse models of amyotrophic lateral sclerosis Type 8. A loss of motor neurones in *VapB^P56S/+^* and *VapB^P56S/P56S^* animals is indicated by a reduction in the number of large choline acetyl transferase–staining cells in the spinal cord. *VapB^−/−^* animals exhibit a relative increase in cytoplasmic TDP-43 levels compared with the nucleus, but no large protein aggregates. Concomitant with these spinal cord pathologies *VapB^P56S/+^*, *VapB^P56S/P56S^* and *VapB^−/−^* animals exhibit age-dependent changes in paw placement and exerted pressures when traversing a CatWalk apparatus, consistent with a somatosensory dysfunction. Extramotor dysfunction is reported in half the cases of motor neurone disease, and this is the first indication of an associated sensory dysfunction in a rodent model of amyotrophic lateral sclerosis. Different rodent models may offer complementary experimental platforms with which to understand the human disease.

## Teaser

Amyotrophic lateral sclerosis Type 8 *VapB^P56S^* rats have reduced large choline acetyl transferase–staining cells in the spinal cord. In *VapB^−/−^* rats, TDP-43 is redistributed to the cytoplasm without aggregation. CatWalk analysis of *VapB^P56S/+^*, *VapB^P56S/P56S^* and *VapB^−/−^* animals detects age-dependent changes in paw placement and exerted pressures consistent with somatosensory dysfunction.

## Graphical Abstract

**Graphical Abstract:**
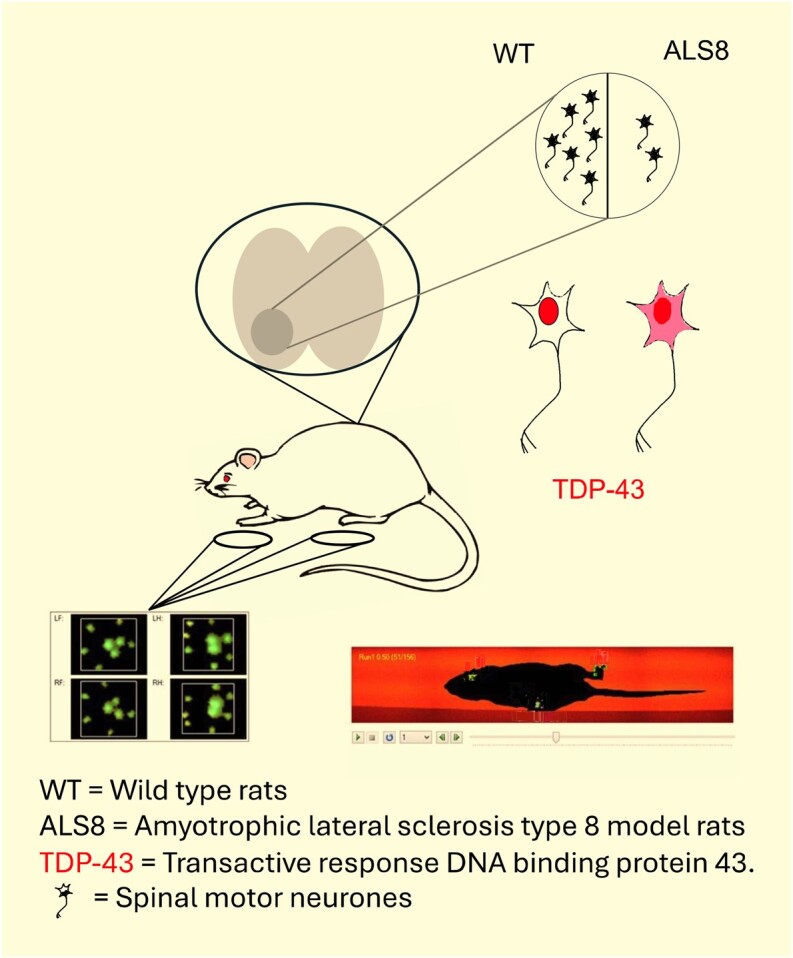

## Introduction

Genetic linkage analysis first identified a missense point mutation in *VAPB* as the cause of a familial human motor neurone disease amyotrophic lateral sclerosis Type 8 (ALS8).^1^ The first cases were reported in Brazil, with others found subsequently in China, Germany, North America and Japan.^2-5^ The originally identified *VAPB^P56S^* mutation is the most frequent and has occurred independently on at least two occasions. Affected Brazilian families can be traced to a single founder who arrived in the country from Portugal in the 16th century.^6^ The *VapB*^P56S^ family in Germany are not related to this founder having a different haplotype.^3,6^ A second disease-associated mutation, *VapB*^T46I^, has also been reported.^7^ Although extremely rare in Europe,^8^ ALS8 *VapB^P56S^* is the most common form of familial motor neurone disease in Brazil.^9^ Patients with ALS8 display different phenotypes, ranging from a typical ALS involving both upper and lower motor neurone dysfunction, to a spinal muscular atrophy.^10,11^ Choking, constipation and sexual dysfunctions indicate autonomic systems can also be affected by the disease.^10^ A well-characterized Chinese ALS8 family also suffer pain in their limbs and lower back.^2^ It is now widely accepted that perhaps as many as 50% of patients with ALS will also suffer extramotor dysfunction, and in ∼10% of cases, the level of cognitive impairment amounts to a frontotemporal dementia.^12^ Cognitive conversion associated with widespread cerebral atrophy has also been recently reported in a German patient with ALS8.^13^ It is not clear how the disease-associated missense mutations disrupt VAPB function leading to neurodegeneration.

The VAMP/synaptobrevin-associated protein (VAP) was first identified in *Aplysia californica* using a yeast two-hybrid screen for VAMP/synaptobrevin-interacting proteins.^14^ Subsequently, homologous proteins were identified in *Drosophila*, rat, mouse and humans.^15-19^ Two separate genes encode the proteins VAPA and VAPB, and a smaller form, VAPC, has been identified as a splice variant of the *VapB* transcript.^15^ The primary structure of VAPA and VAPB is very similar, encompassing an N-terminal domain highly homologous to the major sperm protein (MSP) of *Caenorhabditis elegans*, a central coiled-coil region and a short C-terminal membrane anchor.^19-22^ Both proteins are ubiquitously expressed and enriched on the outer surface of the endoplasmic reticulum (ER).^14,15,17,19^ Individually or in combination, VAPA and VAPB have been suggested to have at least three distinct functions. They serve as docking points to localize endogenous and exogenous proteins to the surface of the ER^23-27^ and mediate interactions of the ER membrane with microtubules and other organelles, such as mitochondria, endosomes and lysosomes.^28^ VAPB facilitates the trafficking of certain plasma membrane proteins through the ER (HCN1/2, Kv2.1/2.2).^29,30^ The N-terminal MSP domain of both proteins is a binding site for proteins containing a ‘two phenylalanines in an acid tract’ or ‘FFAT’ motif.^31^ Both ALS8 mutations lie within the MSP domain of VAPB.^1^ This domain mediates interactions with cytoplasmic proteins, including proteins involved in lipid transport, membrane trafficking, the cytoskeleton and signalling pathways.^23^ The properties of different FFAT motifs are influenced by phosphorylation.^32^

To facilitate further investigations into the role of VAPB in disease and the consequence of the ALS8 P56S mutation, an ALS8 model was recently generated using a new transgenic CRISPR/cas9 method to introduce the *VapB*^P56S^ missense mutation into the rat *VapB* gene.^33^ As part of that work, a *VapB* gene deletion animal was also produced. This report is an initial characterization of these animals to establish their potential utility as a disease model for ALS8 and degeneration in general.

## Materials and methods

### Animals

*VapB^P56S^* and *VapB^−/−^* animals were derived as described.^33^

All procedures were carried out in alignment with the UK Animals (Scientific Procedures) Act 1986, and in accordance with personal and project licences issued by the UK Home Office, and overseen by the University of Edinburgh Animal Welfare and Ethical Review Body. Animals were kept on a 12 h dark–light cycle with food and water available *ad libitum*. Histological analyses were done on six wild type (WT; three males, three females), seven *VapB^P56S/+^* (four males, three females), five *VapB^P56S/P56S^* (three males, two females) and five *Vap^−/−^* (two males, three females).

### Genotyping

DNA was extracted from ear clips taken to identify individual pups at weaning using the ChargeSwitch™ gDNA Micro Tissue Kit (Thermo Fisher). Genotype-specific polymerase chain reaction (PCR) products were generated using primers TGTGGTTCTGTGGAAGCAAG and AGTGTGGTACCCGAGGTGAG. PCR programme 96°C for 3 min then 35 cycles of 30 s at 96°C, 1 min 60°C and 45 s at 72°C. Final elongation of 3 min at 72°C.

### Immunoblot

Tissue was harvested from freshly euthanized animals and homogenized in 20 mM HEPES pH 8.0, containing cOmplete™ protease inhibitor (Roche). Protein concentration was measured spectrophotometrically using Coomassie Plus (Pierce™, Thermo Fisher), and stored at −20°C or −80°C. Homogenates were adjusted to 2 mg/ml in sodium dodecyl sulfate sample buffer, and 20 μg of protein was separated on 12% or 4–12% acrylamide NuPAGE™ Tris-Gycine gels. Proteins were transferred to 0.75 µm polyvinylidene fluoride membrane at 100 V in cold NuPAGE™ transfer buffer (Thermo Fisher) for 1 h. Membranes were then blocked in 5% (w/v) non-fat milk, Tris-buffered saline (TBS) containing 0.1% (v/v) Tween 20 (TBST) for 1 h and left to incubate in primary antibody overnight at 4°C. Immunoreactivity was detected by donkey anti-rabbit horseradish peroxidase-conjugated secondary antibody (1:5000, Jackson ImmunoReseach 711-035-152) and enhanced chemiluminescence (Pierce ECL Western blotting substrate).

### Antibodies

The antibodies used in this study were as follows: rabbit anti-VAPA, as previously reported^17^; #38 rabbit sera raised against bacterially expressed full-length mouse VapB; #6 rabbit sera raised against bacterially expressed MSP domain (aa 1–128) mouse VapB (Murage, PhD thesis, University of Edinburgh, 2022); rabbit anti-human TDP-43 (10782-2AP ProteinTech); goat anti-human ChAT (AB144P Sigma-Aldrich).

### Histology and staining

Tissue was dissected from animals following transcardial perfusion with cold phosphate-buffered saline followed by 4% (w/v) paraformaldehyde, and post-fixed in 4% (w/v) paraformaldehyde for 12 h. Tissue was processed in a Sakura Tissue-Tek TEC 6 processor, embedded in paraffin and stored at room temperature. Sections of L3–L5 spinal cord were cut at 10 µm thickness on a Leica-RM2245 microtome, mounted onto SuperFrost Plus™ slides using a 40°C water bath, dried for 12 h at 60°C and stored at room temperature. Prior to immunostaining, slides were de-waxed by immersion in xylene and decreasing concentrations of methanol and re-hydrated in running water for 5 min. Samples were prepared from six WT (three males, three females), seven *VapB^P56S/+^* (four males, three females), five *VapB^P56S/P56S^* (five males, two females) and five *Vap^−/−^* (two males, three females).

### Immunostaining

Rehydrated slide-mounted tissue sections were incubated in 10 mM sodium citrate buffer (pH 6.0) and heated in a microwave at 400 V for four times 5 min cycles, to recover epitopes. Slides were then cooled and extensively washed in water. Endogenous peroxide activity was quenched by incubation in 3% H_2_O_2_ (in TBS) for 10 min and followed by 2 × 10 min TBST washes. Tissue was blocked for 1 h at room temperature with 4% (v/v) normal goat serum in TBST or bovine serum albumin. Primary antibodies were diluted in the same buffer. Unbound antibody was removed by 3 × 10 min TBST washes, and immunoreactivity detected with labelled secondary antibodies using VECTASTAIN® ABC-HRP Kit or fluorescent anti-sera from Jackson ImmunoResearch; rabbit IgG, PK-4001 for TDP-43 (1:2000); polyclonal rabbit anti-goat #E0466 Dako for ChAT144P (1:100). For all sections, coverslips were mounted using VECTASHIELD® HardSet™ Antifade Mounting Medium (H-1400-10, Vector Laboratories) and stored in the dark at 4°C.

### Image capture and analysis

Brightfield images were captured with a Hitachi HV-F202SCL camera, and fluorescence images with a Hamamatsu Arca Flash camera. Images were rendered using QuPath^34^ and processed in FIJI/ImageJ.^35^ Image analysis for the quantification of large ChAT-immunopositive cells in the spinal cord was done using the automatic threshold ‘minimum’ algorithm tool in ImageJ/FIJI to capture particles with areas between 700 and 2500 μm^2^. For TDP-43 localization analysis, to avoid bias when identifying and outlining nuclei, images were converted to 8-bit, thresholded automatically using the in-built ‘minimum’ algorithm and the ‘analyse particles’ function used to identify nuclei >100 μm^2^. A mask of these positively identified nuclei was generated for each region of interest and resized where necessary to encompass whole nuclei. Nuclear masks were then dilated 10 times to gain a mask of the nucleus and perinucleus. Nuclear and dilated masks were used with FIJI’s ‘measure’ tool to quantify the area and mean intensity of TDP-43 staining in the nucleus and perinucleus (see below equation). Mean intensity values were used to calculate a ratio of nucleus:cytoplasm TDP-43 staining. The log_10_ nucleus:cytoplasm ratio was used to account for exponential scaling of the nucleus:cytoplasm ratio that occurs when nuclear mean intensity is greater than cytoplasmic intensity.


$$\begin{array}{rl} & \mathrm{Perinuclear}\;\mathrm{mean}\;\mathrm{intensity}\;=\\ & \frac{\begin{array}{r} \left(\mathrm{Dilated}\;\mathrm{mean}\;\mathrm{intensity}\;\times \;\mathrm{Dilated}\;\mathrm{area})-(\mathrm{Nuclear}\;\mathrm{mean}\;\mathrm{intensity}\times \mathrm{Nuclear}\;\mathrm{area}\right) \end{array}}{\mathrm{Dilated}\;\mathrm{area}-\mathrm{Nuclear}\;\mathrm{area}} \end{array}$$

### CatWalk

CatWalk apparatus was as described.^36,37^ The room was dimly lit, and the walkway data collection parameters were minimum run duration of 0.5 s, maximum run duration of 10 s, maximum allowed speed variation 60%, camera gain 28.2, intensity threshold 0.23, abort runs after 10 s, label assigned to paw when maximum intensity is >100.

The day before data collection, animals were habituated in their home cage in the behaviour room for 30 min and then allowed three practice runs. Software automatically started capturing data once the rat was visible in the pre-defined run capture area. Recording was aborted if the animal did not run to the end of the capture area within the maximum run time set at 10 s. The practice runs were completed with 5 min breaks in the home cage between runs. On test day, rats were habituated to the low light for 30 min. Data for 10 runs were collected with a 5 min break between runs. After collecting all runs, body weight was recorded, and animals were returned to their housing units. Parameters were as defined by the manufacture. The full data set of the CatWalk analysis done for the study will be made available on request.

### Statistical analysis

Parametric statistical analyses were performed where data were normally distributed, the assumption of normality was violated and non-parametric alternatives were used. Three or more groups defined by a single independent variable were compared by one-way ANOVA or non-parametric Kruskal–Wallis tests. Three or more groups defined by two independent variables in which repeated measures of the dependent variable were made were compared by two-way repeated measure (RM) ANOVA tests. Multiple comparisons adjusted *post hoc* tests were used to determine which groups differed when main effects were significantly different in one-way and two-way ANOVA tests; the exact *post hoc* test used is detailed when used. When the interaction effect in a two-way ANOVA proved statistically significant, simple effect multiple comparisons adjusted *post hoc* tests were performed (effect of one independent variable within one level of the second independent variable) to determine which groups differed. All statistical analyses were performed using GraphPad Prism 10.0.3 for Mac. Sample size (*n*) denotes the number of independent animals. Data are presented as mean ± standard error of mean. Results were deemed statistically significant, where *P* < 0.05.

## Results

### Reduction of VAPB protein in both P56S mutant and knockout animals

**Figure 1:**
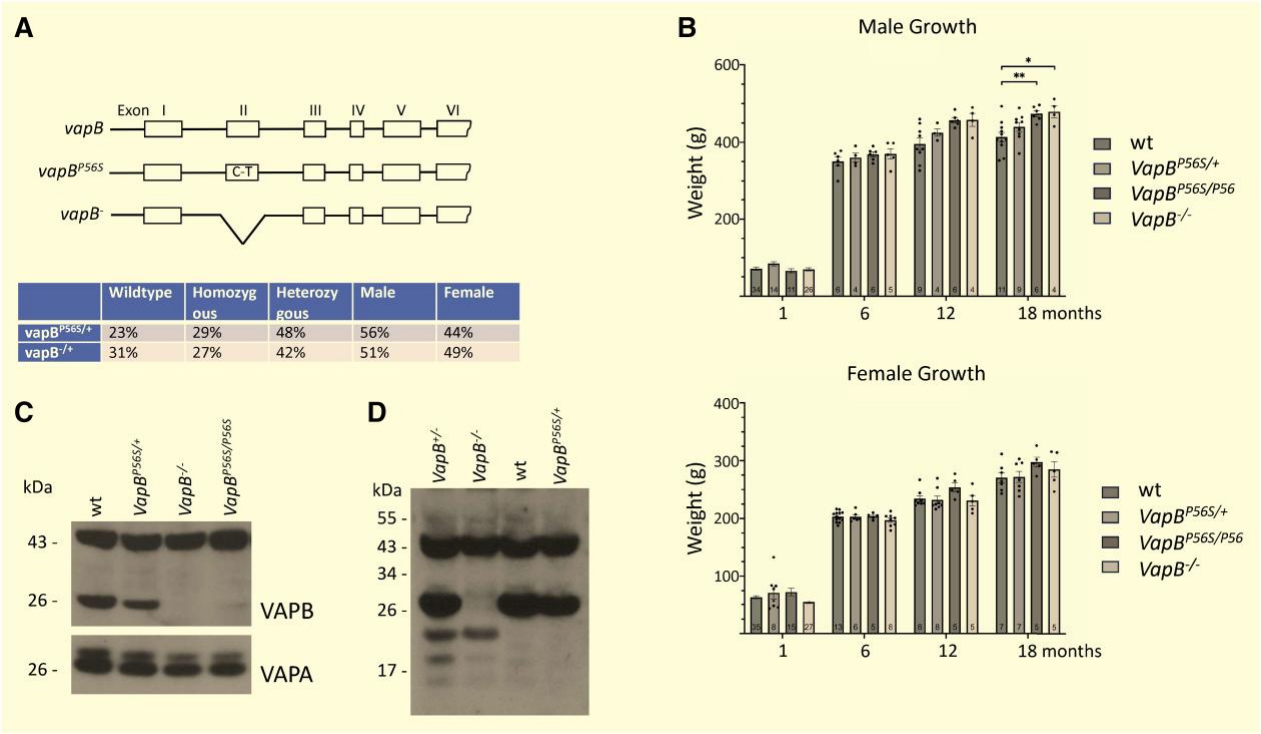
**Genotype frequency and growth rates of *VapB* mutant rats.** (**A**) Schematic of genotypes used in the study. *VapB^P56S/+^* and *VapB^−/+^* colonies were bred as heterozygous crossings. No significant difference was seen in the number of male and females and the relative frequency of homozygous and heterozygous animals followed the expected Mendelian pattern. (**B**) Immunoblot indicates VapB protein levels are reduced to ∼65% of WT in the brains of *VapB^P56S/+^* animals (64.9%, SD 6.4%, *n* = 5), and dramatically reduced in *vapB*^P56S/P56S^ [7.1%, SD 2.9%, *n* = 3 (1 male, 2 females), WT *n* = 4 (2 males, 2 females)]. No full-length VAPB is detected in *VapB*^−/−^ brain. (**C**). Long exposure of immunoblots, including *VapB*^−/+^ samples, reveals a low level of smaller molecular weight proteins in the *vapB*^−/−^ animals that may represent the products of transcripts lacking Exon 2 or other alternatively spliced transcripts. Signal at 45 kDa cross-reacting signal serves as a loading control. Anti-sera #6 and #38 used for (**B**) and (**C**), respectively. See Supplementary Fig. 2 for uncropped immunoblots. (**D**) Body weight of female animals for all *VapB* genotypes was not significantly different from WT animals up to 18 months of age [Kruskal–Wallis test; *H*(3) = 3.858, *P* = 0.2772]. By 18 months of age, genotype significantly affected body weight [Kruskal–Wallis test; *H*(3) = 12.85, *P* = 0.005]. Male *VapB^P56S/P56S^* and *VapB^−/−^* animals were significantly heavier than WT animals (Dunn’s multiple comparisons test; *VapB^P56S/P56S^ P* < 0.01, *VapB^−/−^ P* < 0.05). For all genotypes, *n* ≥ 4, *P < 0.05, **P < 0.01.

The introduction of the ALS8 *VAPB^P56S^* point mutation to the rat *VapB* using a CRISPR/Cas9 approach was previously reported^33^ (Fig. 1A). The C-T base substitution in Exon 2 together with flanking *loxP* sites was introduced to F344 rat gene creating *VapB^P56S/+^* animals. That study also produced lines in which 210–211 bp between the gRNA-targeted sites was deleted, effectively removing Exon 2 and creating a knockout allele, *VapB^−/+^*. Both strains were maintained as heterozygous breeding pairs. Litter sizes were similar, and all predicted genotypes were produced at a frequency not significantly different from those expected form Mendelian inheritance, indicating little or no effect of either mutation on embryo viability (Fig. 1A).

Immunoblot analysis of brain tissues indicate that VABB immunoreactivity in *VapB^P56S/+^* animals is 65% that of WT animals [*n* = 5 animals, standard deviation (SD) 6.4%], and 7% in *VapB^P56S/P56S^* animals (*n* = 3, SD 2.9%). This indicates that the mutant protein does not accumulate to the same levels as WT. The *VapB^−/−^* animals produce no detectable protein of the expected molecular weight (Fig. 1B). Longer exposure of immunoblots reveals low levels of a lower molecular weight protein that is most likely the result of aberrant processing or translation from the Exon 2Δ RNA transcript. Removal of the coding sequence of Exon 2 reduces the predicated molecular weight of the protein by 5.5 kDa (Fig. 1C).

### Increase in body weight at 18 months of age for male rats lacking VAPB protein

Up to 12 months of age, the growth rates of WT, *VapB^P56S/+^*, *VapB^P56S/P56S^* and *VapB^−/−^* animals were similar. However, in the male animals, there was a clear trend for all *VapB* genotypes to be heavier than WT, and this became significant by 18 months of age (Fig. 1D). There was no similar trend or effect in the female animals.

### Loss of lower motor neurones in mutant *VapB* animals

**Figure 2:**
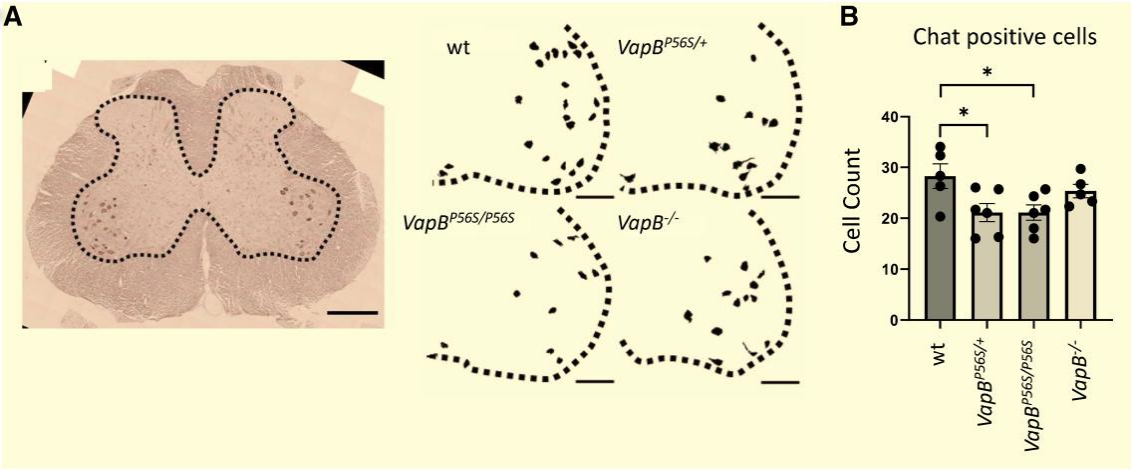
**Loss of spinal motor neurones in the ALS8 rat**. At 18 months, there is a small reduction in ChAT-positive neurons in the lumbrical spinal cord of *VapB^P56S/+^* and *VapB^P56S/P56S^*. (**A**) Ten micrometre sections from L3–L5 spinal cord were immunostained for ChAT. Threshold values selected structures in the ventral horn of between 700–2500 μm^2^. (**B**) The number of large ChAT immunopositive cells was reduced in *VapB*^P56S/+^ and *VapB*^P56S/P56S^. (One-way ANOVA; *F*_3,18_ = 3.817, *P* = 0.0281; Dunnett’s multiple comparisons test; *VapB^P56S/+^ P* < 0.05, *VapB^P56S/P56S^ P* < 0.05.) Three sections were counted and averaged from each animal, *n* = 5–6 animals per genotype (WT, 3 males, 2 females. *VapB^P56S/+^*, 3 males, 3 females. *VapB^P56S/P56S,^* 3 males, 3 females. *VapB^−/−,^* 2 males, 3 females) *P < 0.05. Scale bar on image 500 µm. Scale bar on threshold analysis 200 µm.

ALS8 is a late on-set disease with progressive motor dysfunction in the limbs, trunk and bulbar system.^10^ The majority of ALS8 cases are characterized as a spinal muscular atrophy, lacking pronounced upper motor neurone involvement. To investigate whether the *VapB* mutant animals showed signs of lower motor neuron loss, the number of large choline acetyl transferase (ChAT)-immunopositive cells were quantified in the lumbar spinal cord of 18-month-old WT, *VapB^P56S/+^*, *VapB^P56S/P56S^* and *VapB^−/−^* animals (Fig. 2). There was a significant reduction of ChAT-positive cells in *VapB^P56S/+^* and *VapB^P56S/P56S^* animals compared with WT. No significant change was detected in the *VapB^−/−^* animals.

### Increased cytoplasmic TDP-43 staining in motor neurones lacking VAPB protein

**Figure 3:**
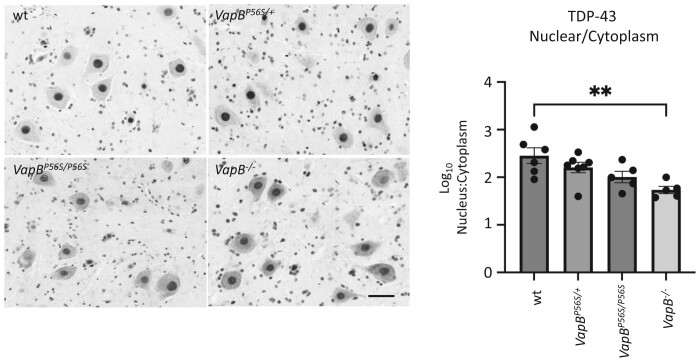
**Increased cytoplasmic TDP-4 3 staining in motor neurones from *VapB^−/−^.*** Sections from the lumbar region of the spinal cord of 18-month-old *VapB* animals were immunostained for TDP-43. Signal intensity in nuclear and perinuclear regions was measured and used to determine the ratio of nucleus to cytoplasmic TDP-43. A relative increase in cytoplasmic TDP-43 was detected in the *VapB^−/−^* animals. No large TDP-43 aggregates were detected in the cytoplasm. One-way ANOVA; *F*_3,19_ = 5.613, *P* = 0.0063; Dunnett’s multiple comparisons test. Three sections containing at least six cells were analysed per animal. *n* = 5–7 animals per genotype (WT, 3 males, 3 females. *VapB^P56S/+^*, 4 males, 3 females. *VapB^P56S/P56S^,* 3 males, 2 females. *VapB^−/−,^* 2 males, 3 females). +/−, standard error. **P < 0.01. Scale bar 50 μm.

TDP-43 pathology in the form of protein aggregation and redistribution from the nuclear to the cytoplasmic compartment is seen in the large majority of ALS cases.^38,39^ Sections from the lumbar region of the spinal cord of *VapB* animals were immunostained for TDP-43. No TDP-43-immunoreactive protein aggregates were detected in the large motor neurons of any of the VapB mutant animals. However, there was a significant increase in the cytoplasmic level of the protein compared with that in the nucleus in *VapB^−/−^* animals as indicated by a reduction in the ratio of the nucleus to cytoplasmic staining (Fig. 3).

### Alteration to paw placement in rats lacking VAPB protein

**Figure 4:**
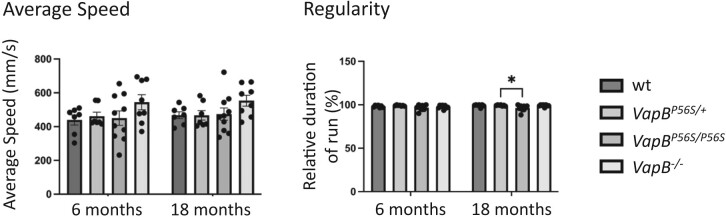
**CatWalk analysis of *VapB* mutant rats.** There was no difference in the average speed at which animals traversed the CatWalk runway. Neither genotype, age nor their interaction had a significant effect upon the average speed at which the animals traversed the CatWalk runway (two-way RM ANOVA; *F*_3,28_ = 2.105, *P* = 0.12; *F*_1,28_ = 0.8333, *P* = 0.37; *F*_3,29_ = 0.6027, *P* = 0.97). *VapB* animals had normal regularity of gait at 6 months but by 18 months a slight deficit was detected in *VapB^P56S/P56S^* animals. Genotype significantly affected regularity (two-way RM ANOVA; *F*_3,28_ = 6.292, *P* = 0.002) with 18-month-old *VapB^P56S/P56S^* animals showing significantly reduced regularity compared with WT animals (Dunnett’s multiple comparisons test; *P* < 0.05).

**Figure 5:**
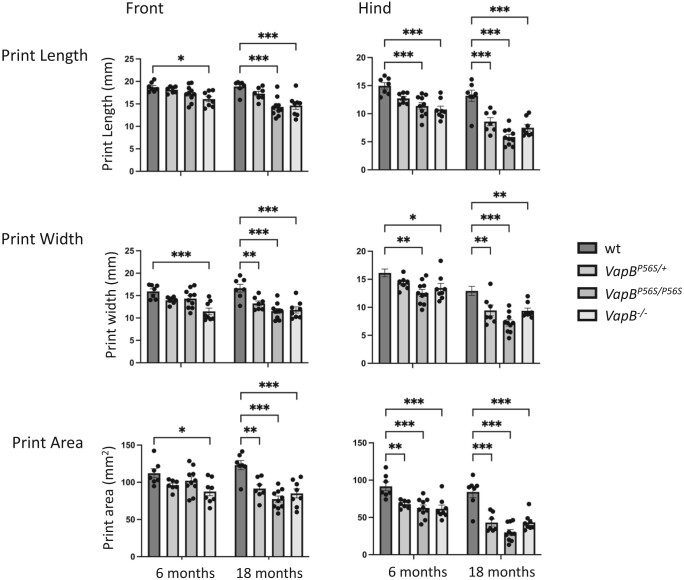
**Paw print size parameters decreased progressively over 18 months.** There was a statistically significant interaction between the effects of age and genotype on both front and hind paw print length (two-way RM ANOVA; front *F*_3,28_ = 4.639, *P* = 0.009 | hind *F*_3,28_ = 6.088, *P* = 0.003). Main effects analysis reveals that front paw length is significantly reduced in *VapB^−/−^* animals relative to WT at 6 and 18 months (Dunnett’s multiple comparisons test; 6 months *P* < 0.01, 18 months *P* < 0.001) with *VapB^P56S/P56S^* animals only differed at 18 months (*P* < 0.001). Similar main effects analysis reveals that hind paw length is significantly reduced relative to WT in *VapB^−/−^* and *VapB^P56S/P56S^* animals at 6 and 18 months (all *P* < 0.001), while only significant at 18 months in *VapB^P56S/+^* animals (*P* < 0.001). A similar pattern was seen for paw print width and total paw print area.

**Figure 6:**
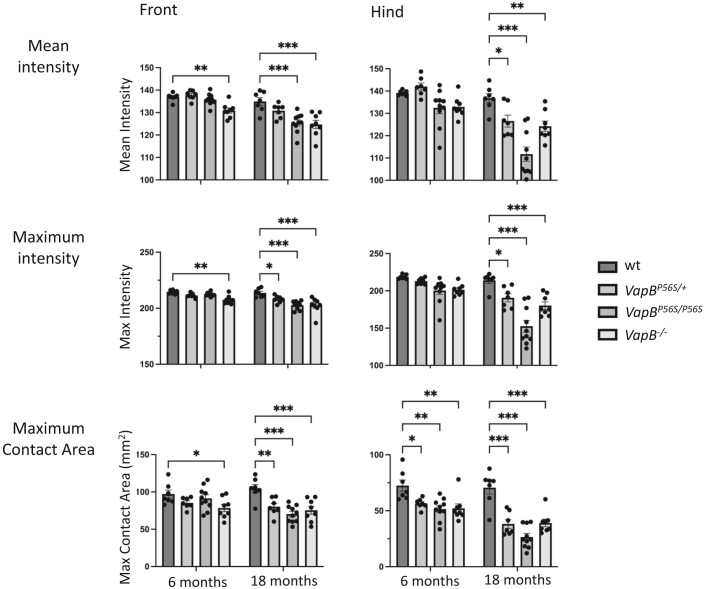
**Intensity reflects the pressure exerted on the CatWalk through the paws.** Both the mean and maximum intensity values were reduced in the front and hind paws of *VapB* animals over 18 months compared with WT animals. There was a statistically significant interaction between the effects of age and genotype on both front and hind paw print mean intensity (two-way RM ANOVA; front *F*_3,28_ = 5.187 *P* = 0.006 | hind *F*_2,28_ = 5.708 *P* = 0.004) and maximum intensity (two-way RM ANOVA; front *F*_3,28_ = 6.314 *P* = 0.002 | hind *F*_3,28_ = 7.405 *P* < 0.001). Main effect analysis reveals that front paw mean intensity is significantly reduced relative to WT in *VapB^−/−^* animals at 6 and 18 months (Dunnett’s multiple comparison test; 6 months *P* < 0.01, 18 months *P* < 0.001) but only by 18 months for *VapB^P56S/P56S^* animals (*P* < 0.001). Significant differences in hind paw mean intensity relative to WT were only observed at 18 months in *VapB* animals (P56S/+ *P* < 0.05, P56S/P56S *P* < 0.001, −/− *P* < 0.01). A similar pattern was observed for maximum paw print intensity; font paw maximum intensity is significantly reduced *VapB^−/−^* animals at 6 months (*P* < 0.001) and 18 months (*P* < 0.001) but only at 18 months for *VapB^P56S/P56S^* (*P* < 0.001) and *VapB^P56S/+^* animals (*P* = 0.006); hind paw maximum intensity is significantly reduced in *VapB^P56S/P56S^* animals at 6 months (*P* < 0.05) with all VapB genotypes significantly differing from WT at 18 months (P56S/+ *P* < 0.05, P56S/P56S *P* < 0.001, −/− *P* < 0.01). Ten runs were analysed and average from each animal at each time point, *n* = 7–10 animals per genotype (WT, 4 males, 3 females. *VapB^P56S/+^*, 4 males, 3 females. *VapB^P56S/P56S^*, 5 males, 5 females. *Vap^−/−^*, 5 males, 4 females). *P < 0.05, **P < 0.01, ***P < 0.001.

The motor function of the *VapB* mutant rats was tested using an automated CatWalk gait analysis, as they aged over an 18-month period.^36,37^ This analysis provided quantitative measures of ambulatory speed, gait, limb coordination as well as placement and pressure of paws. The apparatus illuminate’s footprints and pressure are then transformed into green fluorescence with brightness directly correlating to contact intensity. Compared with WT animals, there were no significant changes in basic speed of the *VapB^P56S/+^*, *VapB^P56S/P56S^* or *VapB^−/−^* animals (Fig. 4). The gait regularity is normal at 6 months, but at 18 months, a slight deficit is detectable in *VapB^P56S/P56S^*. There were significant changes in the paw placement characteristics consistent with a decrease in paw pressure during walking. This is despite the increased weight of the mutant male animals. Compared with WT animals at 6 months, the paw print length, width and total area for the front limbs was significantly decreased in *VapB^−/−^* animals. This effect progressed, and by 18 month it became significant in all *VapB* mutant animals. The effect on the hind limbs of all genotypes was earlier and significant changes in width and area of paw print were detected at 6 months of age (Fig. 5). The force exerted by the paws is indicated by the mean and maximum intensity measure (Fig. 6). Again, the effect was more pronounced in the hind limbs, and at 18 months, all *VapB* animals exerted significantly less pressure on the surface compared with the WT animals.

It is not possible to make direct comparisons between parameters measured by the CatWalk in rodents and the motor dysfunction suffered in patients with ALS. However, these changes are progressive and appear to correlate with VAPB expression levels, supporting the conclusion that mutations in *VapB* compromise motor performance in the rat in an age-dependent fashion.

## Discussion

This study conclusively demonstrates that mutations in *VapB* compromise motor performance in the rat in an age-dependant and progressive fashion. It is the first time that changes to paw placement have been reported in an animal model of ALS and may indicate sensory dysfunction, such as pain sensitivity could be recapitulated in the mutant *VapB* rats.^40^ Fifty per cent of all patients with ALS suffer extramotor dysfunction, mainly relating to the sensory and autonomic systems,^41^ and pain is reported by the majority of patients with ALS8.^2^

VAPB levels in brain tissue are reduced in *VapB^P56S/+^* animals, and barely detectable in homozygote mutants. Reduced levels of VAPB have been detected in sporadic non-familial ALS cases,^42^ and its levels are 50% of normal in motor neurones generated from induced pluripotent stem cells of patient with ALS8.^43^ Furthermore, *VapB* knockout mice exhibit mild motor defects with a decreased cage hang time at 18 months of age.^44^ These results suggest that VAPB insufficiency may play a role in ALS8 pathology. A similar reduction in VAPB expression was seen in knock-in mice but was, in part, attributed to increased levels of insoluble protein.^45^ We detected no difference in solubility of VAPB protein from the *VapB^P56S/+^* animals compared with WT [1% NP40 (v/v), Supplementary Fig. 1]. The reported decreased VAPB levels in spinal cord of sporadic non-familial cases is consistent with the suggestion that VAPB insufficiency may contribute or is associated with the disease process.^42^ However, the most profound changes in paw placement occur at 18 months in the *VapB^P56S/P56S^* animals, which would be consistent with a dominant negative function of the mutant protein in combination with a pathological insufficiency.

Male *VapB^P56S/P56S^* and *VapB^−/−^* animals are significantly heavier than WT animals by 18 months. This was not seen in the females of the same genotypes. Gender-specific differences are reported for many neurodegenerative diseases.^46^ ALS is slightly more common in males,^47^ and some evidence suggest females are more sensitive to environmental factors that may influence ALS frequency and prognosis, such as smoking.^48^ The muscle wasting occurring in motor neurone disease (MND) can be accompanied by a reduction in body mass index (BMI).^49^ However, it is unclear whether body weight loss is a direct consequence of the disease or the result of swallowing and eating difficulties. When sufficient food can be consumed the BMI of patients can actually increase.^50^ The weight gain seen in older *VapB*^P56S/P56S^ and *VapB*^−/−^ male animals was not seen in the knock-in mouse model,^45^ and weight loss was seen in one of the over-expressing transgenic animals.^51^ However, significant weight gain was seen in both male and female TDP-43^Q331K^ transgenic and knock-in TDP-43^Q331K^ mice.^52,53^ The mechanistic relationship between weight gain and causative ALS mutations is not clear, but it is notable that hypothalamic dysfunction has been reported in the SOD-1^G93A^ mouse.^54^ Interestingly, it has been suggested that BMI may preferentially increase in patients with cognitive deficits.^55^

Several rodent models for ALS8 based on the P56S mutation have been generated. Of these, three were transgenic approaches to over express the WT and mutant forms of human VAPB under different ubiquitous high-activity promoters.^51,56-58^ The expression of VAPB^P56S^ from the endogenous rat gene produces phenotypes distinct from over expression of human proteins in transgenic mice. In VAPB^P56S^ transgenic over-expressing mice, protein aggregates of VAPB and ubiquitin were present in the cytoplasm of motor neurones. TDP-43 pathology was induced in some cases.^56^ In two transgenic lines, stress responses and structural changes in the ER were observed but in only one line, significant motor function deficit was reported.^51,57^ Aliaga *et al*.^51^ reported a reduced number of cortical motor neurones but no change in spinal motor neurones.

The ALS8 *VapB^P56S^* knock-in rat characterized here exhibits a similar but distinct phenotype compared with the previously reported *VapB^P56S^* mouse strain.^45^ In the rat, a loss of spinal motor neurones was observed, and there was a redistribution of TDP-43 to the cytoplasm without overt protein aggregation. In contrast, in the mouse, no loss of motor neurones was reported, and there was TDP-43 aggregation pathology.^45^ Therefore, these animal models represent complementing systems with which to explore the consequences of VAPB^P56S^ expression and, therefore, the potential pathological mechanisms of the human disease ALS8.

The increase in cytoplasmic TDP-43 correlates with the motor behavioural changes and the progressive loss of VAPB levels. Cytoplasmic accumulation of TDP-43 is sufficient to cause neuronal death when induced in mouse hippocampus and forebrain.^59^ On the contrary, in the current study, increased levels of cytoplasmic TDP-43 do not correlate with the loss of motor neurones, suggesting it may not be sufficient to trigger cell death. Or, that an alternative pathological response is responsible for the cell loss, where the translocation of TDP-43 is more toxic in combination with VAPB^P56S^. It also suggests that in the rat VAPB dysfunction may lead to disruption of TDP-43 nuclear transport, but may not induce the post-translational modifications associated with aggregation and accumulation in stress granules.^60^ Changes in nuclear transport are seen in SOD1 mice^61^ and *Drosophila C9orf72* models.^62,63^ Similarly, reduced nuclear TDP-43 and Ran-dependent nuclear transport have been reported in the fibroblast cultures of patient with ALS8.^4^

The loss of VAPB or the expression of VAPB^P56S^ in the rat leads to subtle changes in the placement of both front and rear paws that increases in severity with age. CatWalk analysis of motor behaviour in rats cannot be directly compared with human studies, but as ALS8 is a late onset disease with a relatively slow progression when compared with other forms of ALS,^10^ it is perhaps not unexpected that the knock-in rodent phenotype is relatively subtle compared with the more profound effects of transgenic overexpression in other ALS models. The heterogeneity of MND can make subtle changes very difficult to detect across patients.^64^ However, automated analysis of kinematics and motor behaviour are very useful to monitor the progression of disease in an individual.^65^ Similar decreases in paw print area have been reported in studies using the CatWalk system to measure the recovery of motor performance following median nerve resection in rats.^66^ Bao *et al*.^67^ reported decreased paw print area following the induction of chronic pain by median nerve compression and suggest it may reflect a response to pain. The surviving patients from the only identified Chinese ALS8 family all reported pain in their extremities.^2^ The rat behaviour indicates that this feature of the human disease may be recapitulated in the rat. Pain is not uncommon in patients with ALS,^68^ but it is unclear whether it results from lack of mobility and cramps or is part of the neurological basis of the disease.

The profound motor phenotypes induced in overexpression animal models may obscure more subtle extrapyramidal deficits, such as those exhibited by the *VapB^P56S^* knock-in animals. Such phenotypes may more closely relate to endogenous pathological processes associated with a particular gene mutation. Elucidating the mechanisms of pathology in an endogenous background may better inform the translation of such work to the human condition. In this instance, the changes in paw placement provides evidence that dysfunction of VAPB affects a motor behaviour and may reflect an additional sensory pathology. Identifying the locus and nature of a sensory dysfunction in the *VapB^P56S^* rats is of basic interest and could suggest new palliative treatments for patients with ALS suffering pain. The identification of different potential pathological mechanisms in experimental systems indicates that a plurality of approaches may be required to capture a comprehensive understanding of the human disease. MND is on a spectrum of disorders ranging from a pure motor neurone degeneration to frontotemporal lobe dementia (FTLD).^69^ The *VapB^P56S^* rats represent a new and distinct model for basic mechanistic research and a potential pre-clinical model for extrapyramidal pathologies in the spectrum of MND/FTLD disease.

## Supplementary Material

fcae184_Supplementary_Data

## Data Availability

Raw data were generated at The Centre for Discovery Brain Science at Edinburgh University. Derived data supporting the findings of this study are available from the corresponding author on request.
