# Supplementary material for: Spinal cord neurone loss and foot placement changes in a rat knock-in model of amyotrophic lateral sclerosis Type 8
Source: Brain Commun. 2024 May 24;6(3):fcae184. doi: 10.1093/braincomms/fcae184 (PMC11154649; doi:10.1093/braincomms/fcae184)
Supplement: fcae184_Supplementary_Data [file fcae184_supplementary_data.pdf]

Supplementary Figure 1

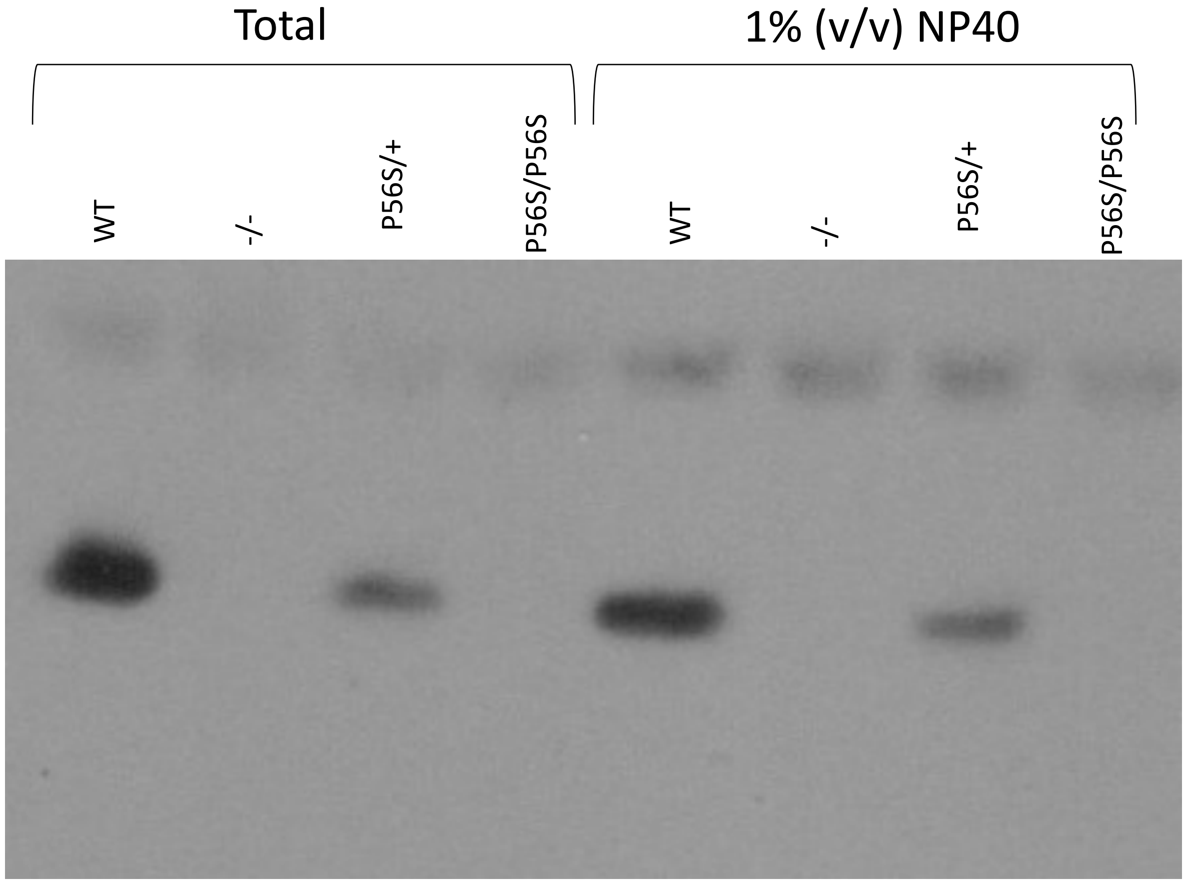

Supplementary Figure 1. VAPB^{P56S} in the rat is not enriched in NP40 insoluble aggregates. Homogenates of brain were extracted with 1% (v/v) NP40 or lysed directly in SDS sample buffer (Total). 25μg of protein per well.

Supplementary Figure 2.

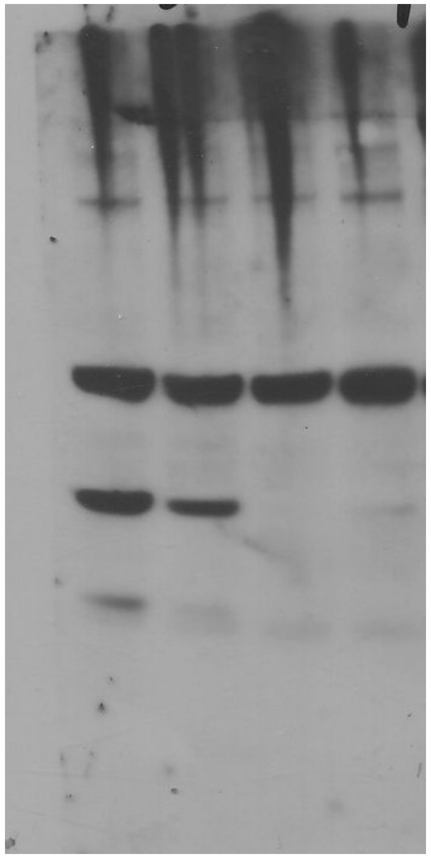

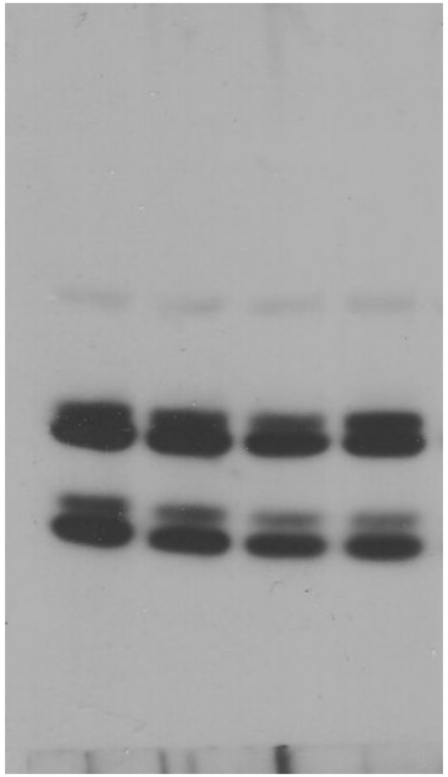

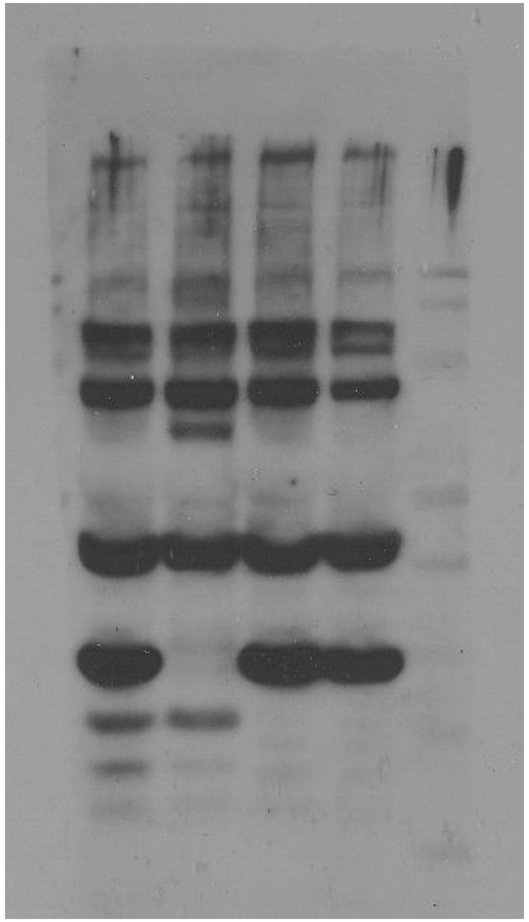

Supplementary Figure 2. Uncropped scans of immunoblots presented in Figure 1.
